# Supplementary material for: Shallow-Water Hydrothermal Vents as Natural Accelerators of Bacterial Antibiotic Resistance in Marine Coastal Areas
Source: Microorganisms. 2022 Feb 21;10(2):479. doi: 10.3390/microorganisms10020479 (PMC8877554; doi:10.3390/microorganisms10020479)
Supplement: Supplementary file 1 [file microorganisms-10-00479-s001.zip › microorganisms-1591602-supplementary.pdf]

Supplementary Materials

Shallow-Water Hydrothermal Vents as Natural Accelerators of Bacterial Antibiotic Resistance in Marine Coastal Areas

Erika Arcadi*, Eugenio Rastelli*, Michael Tangherlini, Carmen Rizzo, Monique Mancuso, Marilena Sanfilippo, Valentina Esposito, Franco Andaloro, Teresa Romeo

This file includes:

Supplementary Tables S1–S2

Supplementary Figure S1

Table S 1. Concentrations of HMs in the sediments collected in this study. Reported are average values (in µg g^{-1}) and SDs.

	"Hot" vent	"Cold" vent	Bottaro Crater
Al	10767 ±1365	9675 ±650	5333 ±351
As	13.4 ±1.5	15.3 ±3.4	87.8 ±64.0
Cd	0.03 ±0.00	0.03 ±0.01	0.04 ±0.01
Cr	5.6 ±0.7	6.4 ±0.5	17.8 ±2.0
Fe	17333 ±577	15250 ±1258	18401 ±11961
Mn	71.8 ±8.9	81.5 ±11.1	550 ±564
Hg	0.06 ±0.01	0.09 ±0.05	0.12 ±0.07
Ni	3.3 ±0.6	3.5 ±0.4	3.2 ±1.7
Pb	7.7 ±0.8	9.0 ±1.2	27.7 ±3.0
Cu	19.1 ±3.4	18.5 ±1.3	6.1 ±4.3
V	61.4 ±6.4	66.1 ±6.0	118.4 ±62.8
Zn	20.5 ±5.0	20.1 ±0.8	38.1 ±13.0

Table S2. List of primers used in this study, with details of gene function, primer sequence, annealing temperature, amplicon size and detailed related references.

Name	Gene function	Primers sequences (5'-3')	Annealing temperatures (°C)	Amplicon size (bp)	References
*ars*B	Arsenite efflux ATP ase	GGTCTATGCGCTGGAGCAATTGAA<br>TGCTGGGCATGTTGTTCATTACCG	46	500	Bouskill et al., 2007
*czc*A	Cobalt/Zinc/Cadmium efflux protein	GTTCACCTTGCTCTTCGCCATGTT<br>ACAGGTTGCGGATGAAGGAGATCA	63	320	Bouskill et al., 2007
*cop*A	Copper translocating ATPase	CGGTCTCTA CGAATACCGCTTCAA<br>GAAATAGCTCATTGCCGAGGCGTT	55	300	Bouskill et al., 2007
*mer*A	Hg reductase	GAAGCGGGTGAACTGATCC<br>TCGTCAGGTAGGGGAACAAC	53	500	Larose et al., 2013
*pbr*T	Pb uptake protein	AGCGCGCCCAGGAGCGCAGCG<br>TCTTGGCTCGAAGCCGTCGAGRTA	55	450	Roosa et al., 2014

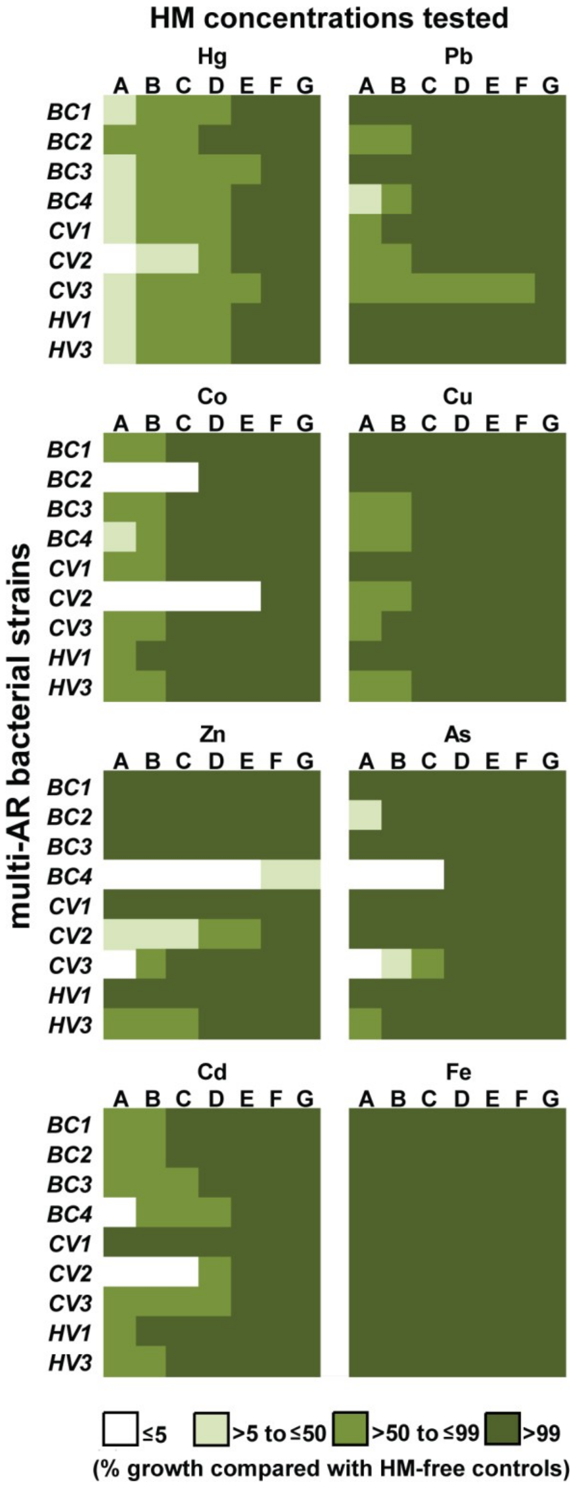

Figure S1. Results of the physiological tests conducted on the multi-AR bacterial strains identified in this study, to assess their tolerance/resistance to different heavy metals. Letters form A to G indicate the different concentrations at which each HM was tested (A=10000 ppm, B=5000 ppm, C=1000 ppm, D=500 ppm, E=100 ppm, F=50 ppm, G=10 ppm). The color legend shows the different growth levels of each strain at each of the concentrations tested, compared with growth in the respective HM-free controls.
